# Monthly Record of Deaths in the City of Buffalo

**Published:** 1858-10

**Authors:** H. D. Garvin

**Affiliations:** Health Physician


### ART. VI.—*Report of Mortality in Buffalo for the Month of Aug.*, 1858. By H. D. Garvin, M. D., Health Physician.

| DISEASES. | No. | Males. | Females. | No Sex given. |
|---|---|---|---|---|
| Accident, | 3 | 2 | 1 | |
| Apoplexy, | 1 | 1 | | |
| Asthma, | 1 | | | 4 |
| Cholera Infantum, | 53 | 30 | 19 | 4 |
| Congestion of Brain, | 6 | 3 | 3 | |
| " " Lungs, | 3 | 1 | 2 | |
| Convulsions, | 27 | 13 | 14 | |
| Coup de Soleil, | 1 | 1 | | |
| Croup, | 3 | | 3 | |
| Dentition, | 2 | 2 | | |
| Diarrhœa, | 18 | 10 | 7 | 1 |
| Drowning, | 8 | 7 | 1 | |
| Dropsy, | 2 | | 2 | |
| Dysentery, | 22 | 9 | 8 | 5 |
| Epilepsy, | 1 | 1 | | |
| Fever, Typhoid, | 5 | 4 | 1 | |
| " Congestive, | 1 | | 1 | |
| Hooping Cough, | 3 | 1 | 1 | 1 |
| Hydrocephalus, | 4 | 1 | 1 | 2 |
| Inflammation of Stomach, | 1 | | 1 | |
| " " Liver, | 1 | | | 1 |
| Intemperance, | 1 | 1 | | |
| Insanity, | 1 | | 1 | |
| Laryngitis, | 1 | 1 | | |
| Marasmus, | 6 | 1 | 5 | |
| Meningitis, | 2 | | 2 | |
| Nursing Sore Mouth, | 1 | | 1 | |
| Old Age, | 8 | 5 | 3 | |
| Paralysis, | 3 | 1 | 2 | |
| Phthisis Pulmonalis, | 16 | 9 | 7 | |
| Pneumonia, | 1 | 1 | | |
| Scarlatina, | 4 | | 3 | 1 |
| Scirrhus of Uterus, | 1 | | 1 | |
| " " Liver, | 1 | 1 | | |
| Scrofula, | 1 | | 1 | |
| Still Born, | 7 | 3 | 3 | 1 |
| Suicide, | 1 | 1 | | |

### REGISTER OF MORTALITY—Continued.

| DISEASES. | No. | Males. | Females. | No Sex given. |
|---|---|---|---|---|
| Syphilis, | 1 | | 1 | |
| Ulceration of Bowels, | 2 | 1 | | 1 |
| Unknown, | 15 | 11 | 3 | 1 |
| Total, | 238 | 122 | 98 | 18 |

### SEXES.

| | |
|---|---|
| Males, | 122 |
| Females, | 98 |
| Sex not given, | 18 |
| Total, | 238 |

### AGES.

| | | | |
|---|---|---|---|
| Still-born, | 7 | Between 20 years and 30 years, | 13 |
| 1 day, | 0 | " 30 " " 40 " | 12 |
| 1 day and 30 days, | 19 | " 40 " " 50 " | 12 |
| Between 1 month and 6 months, | 36 | " 50 " " 60 " | 5 |
| " 6 months and 12 " | 39 | " 60 " " 70 " | 3 |
| " 1 year " 3 years, | 53 | " 70 " " 80 " | 9 |
| " 3 " " 5 " | 11 | " 80 " " 90 " | 2 |
| " 5 " " 10 " | 9 | " 90 " " 100 " | 0 |
| " 10 " " 20 " | 6 | " 100 " | 0 |
| | 180 | | 56 |
| Ages not given, | 18 | | 180 |
| Total, | 238 | | |

### NATIVITIES.

| | | | |
|---|---|---|---|
| American, (colored, 2,) | 187 | Prussian, | 0 |
| German, | 22 | Italian, | 0 |
| Irish, | 17 | French, | 2 |
| English, | 5 | Scotch, | 1 |
| Canadian, | 3 | Switzerland, | 0 |
| Wales, | 1 | Country not given, | 0 |
| Total, | | | 238 |

